# Porous Nanoparticles With Self-Adjuvanting M2e-Fusion Protein and Recombinant Hemagglutinin Provide Strong and Broadly Protective Immunity Against Influenza Virus Infections

**DOI:** 10.3389/fimmu.2018.02060

**Published:** 2018-09-12

**Authors:** Valentina Bernasconi, Beatrice Bernocchi, Liang Ye, Minh Quan Lê, Ajibola Omokanye, Rodolphe Carpentier, Karin Schön, Xavier Saelens, Peter Staeheli, Didier Betbeder, Nils Lycke

**Affiliations:** ^1^Mucosal Immunobiology and Vaccine Center, Department of Microbiology and Immunology, Institute of Biomedicine, Sahlgrenska Academy, University of Gothenburg, Gothenburg, Sweden; ^2^Lille Inflammation Research International Center – U995, University of Lille, INSERM and CHU Lille, Lille, France; ^3^Institute of Virology, University Medical Center Freiburg, Freiburg, Germany; ^4^VIB-UGent Center for Medical Biotechnology, Ghent, Belgium; ^5^Department of Biomedical Molecular Biology, Ghent University, Ghent, Belgium; ^6^Faculty of Medicine, University of Freiburg, Freiburg, Germany; ^7^Faculté des Sciences du Sport, University of Artois, Arras, France

**Keywords:** mucosal vaccination, influenza A virus, CTA1-DD, maltodextrin nanoparticles, targeted adjuvant, nasal immunization, Universal vaccine

## Abstract

Due to the high risk of an outbreak of pandemic influenza, the development of a broadly protective universal influenza vaccine is highly warranted. The design of such a vaccine has attracted attention and much focus has been given to nanoparticle-based influenza vaccines which can be administered intranasally. This is particularly interesting since, contrary to injectable vaccines, mucosal vaccines elicit local IgA and lung resident T cell immunity, which have been found to correlate with stronger protection in experimental models of influenza virus infections. Also, studies in human volunteers have indicated that pre-existing CD4^+^ T cells correlate well to increased resistance against infection. We have previously developed a fusion protein with 3 copies of the ectodomain of matrix protein 2 (M2e), which is one of the most explored conserved influenza A virus antigens for a broadly protective vaccine known today. To improve the protective ability of the self-adjuvanting fusion protein, CTA1-3M2e-DD, we incorporated it into porous maltodextrin nanoparticles (NPLs). This proof-of-principle study demonstrates that the combined vaccine vector given intranasally enhanced immune protection against a live challenge infection and reduced the risk of virus transmission between immunized and unimmunized individuals. Most importantly, immune responses to NPLs that also contained recombinant hemagglutinin (HA) were strongly enhanced in a CTA1-enzyme dependent manner and we achieved broadly protective immunity against a lethal infection with heterosubtypic influenza virus. Immune protection was mediated by enhanced levels of lung resident CD4^+^ T cells as well as anti-HA and -M2e serum IgG and local IgA antibodies.

## Introduction

The quest for a broadly protective influenza vaccine is ongoing. Whereas many different strategies have been employed to design a novel vaccine, a common denominator for these has been to identify conserved viral epitopes that could serve as effective vaccine components (1). Attention has been given to epitopes from the hemagglutinin (HA) stem region in order to raise neutralizing antibodies against conserved structures of the protein (2–6). The prevailing idea is that protective antibodies are largely neutralizing antibodies, but also antibodies acting through antibody-dependent cell-mediated cytotoxicity (ADCC) could prevent disease, as shown in experimental models (7–9). To the latter category of ADCC-acting antibodies we count antibodies against the ectodomain of the influenza A matrix protein 2 (M2e), an ion channel protein which, in fact, is one of the most explored vaccine subcomponents for a universal influenza vaccine today (10–13) M2e as part of a virus-like particle or a fusion protein has been shown to stimulate strong protection against homologous as well as heterologous influenza virus infections in different animal models (14–16). Furthermore, clinical studies have indicated that cell-mediated immune responses, more than antibodies, may be critical for a broadly protective influenza vaccine and, hence, not only M2e, but also several internal structural proteins have been considered for a universal flu vaccine (10, 13). While both memory CD4^+^ and CD8^+^ T cells have been found to correlate with protection against heterosubtypic influenza virus strains, experimental evidence in this regard points to a particularly critical function of lung resident memory T cells for protection (17–20). Most influenza vaccines are injectable vaccines, but these are poor inducers of lung resident memory T cells (13, 21). Therefore, many researchers have focused efforts on mucosal vaccines, which have been found superior to injectable vaccines at stimulating lung resident memory T cells, concomitant with strong secretory IgA (sIgA) and significant systemic IgG immune responses (22).

We have previously developed a universal influenza vaccine candidate by incorporating the M2e-peptide into the non-toxic CTA1-DD adjuvant molecule (16). The CTA1-DD molecule exploits the full immunomodulating ability of CTA1, which is the ADP-ribosylating enzyme from cholera toxin (CT), linked in a fusion protein (FPM2e) that employs the D-fragment from *Staphylococcus aureus* protein A as a cell targeting unit (23–25) CTA1-3M2e-DD was found to strongly protect against a challenge infection with a heterosubtypic influenza A virus strain (H1N1/PR8) (26). Our vaccine adjuvant molecule is lacking the CTB pentamer of CT and cannot bind to the GM1-ganglioside receptors present on most nucleated cells, including nerve cells (27, 28). This way, CTA1-3M2e-DD is completely safe and non-toxic even when given intranasally (i.n) contrary to CT or other GM1-binding toxin adjuvants that can cause facial nerve paralysis, also described as Bell's palsy (29). Interestingly, the CTA1-3M2e-DD not only stimulated strong M2e-specific serum IgG and mucosal IgA antibody responses, but we also identified a critical induction of lung resident M2e-specific memory CD4^+^ T cells (16, 26). We observed that M2e-specific CD4^+^T cells were dominated by Th17 cells, which conveyed protection against influenza that was independent of anti-M2e-antibodies. Accordingly, we believe the CTA1-3M2e-DD, generating both lung resident memory CD4^+^T cells and M2e-specific antibodies, is a good candidate for a broadly protective influenza vaccine.

However, to improve vaccine stability and mucosal delivery of the fusion protein, we sought to explore the combination of the FPM2e with a nanoparticle (30). We used our well established technology to incorporate CTA1-3M2e-DD into porous maltodextrin nanoparticles (NPLs) to further improve the immunogenicity and disease protective functions of the vaccine candidate (31). Apart from shielding the protein against degradation, we speculated that the combined FPM2e:NPL vaccine formulation would facilitate breaching of the mucosal membrane barrier and, in this way, augment antigen uptake in migrating dendritic cells (DC) (32, 33). The positively charged NPLs used in this work have three main components: the reticulated maltodextrin, the anionic lipid (DPPG) and the protein, which are all linked together by non-covalent interactions (Van der Waals forces and electrostatic interactions). Hence, the NPL hosts a negative hydrophobic core surrounded by a positively charged polysaccharide shell (34). We have reported previously that nasal immunizations with similar NPL preparations could stimulate significant protection against Toxoplasma *gondii* in mice (35, 36). An additional advantage of the NPL technology is that it allows for loading of multiple proteins in the same particle. This gave us the opportunity to explore whether anti-influenza protection could be improved with NPLs that carry both the CTA1-3M2e-DD and recombinant HA. Thus, the present study was undertaken to investigate whether the combined HA:FPM2e:NPL vaccine vector, hosting the CTA1-3M2e-DD and recombinant HA, stimulated enhanced protective immunity against influenza virus infections. A special focus was given to the uptake and antigen-processing of the combined vector by DCs, which are the essential primers of CD4^+^ T cell immunity (37).

## Materials and methods

### Mice and immunizations

Age- and sex-matched BALB/c, C57BL/6 or DBA/2 mice were obtained from Harlan (The Netherlands) or Janvier Laboratories (France). The Eα-specific T cell receptor transgenic B6.Cg-Tg(Tcrα,Tcrβ)3Ayr/J mice were obtained from The Jackson Laboratories (USA). Mice were maintained under specific pathogen-free conditions at the Laboratory for Experimental Biomedicine (EBM) (University of Gothenburg, Sweden) or at the Laboratory of Virology (University of Freiburg, Germany). Experiments were ethically approved by local committees regulating animal ethics at the universities of Gothenburg and Freiburg, respectively. A single or three immunizations with 10 days between immunizations were given intranasally (i.n) to 4–6 weeks old mice. As indicated, an i.n antigen dose of 1 or 5 μg of protein was given in a volume of 20 μl i.n to each mouse. Mice were sacrificed after 1–2 weeks following the final immunization or virus challenge infection and spleens, mediastinal lymph nodes (mLN), serum, and broncheoalveolar lavage (BAL) were collected. Serum and BAL were taken at times indicated and stored at −20°C until further analyzed.

### Fusion protein construction

CTA1(C189A)-3M2e-DD, with enzymatic activity, CTA1(R9K)-3M2e-DD, the enzymatically inactive mutant, CTA1-DD and CTA1(C189A)-3Eα-DD were produced in *E. coli* by MIVAC Development AB, Sweden, as previously described (16). The first two constructs carry three copies of the extracellular domain of the influenza virus M2 protein (SLLTEVETPIRNEWGSRSNDSSD) derived from the A/Victoria/3/75 (H3N2) virus strain. CTA1(C189A)-3Eα-DD carries 3 copies of the Eα 52-68 peptide (ASFEAQGALANIAVDKA). The fusion proteins were routinely tested for the presence of endotoxin, using the limulus amebocyte lysate assay (LAL Endochrome TM Charles River Endosafe, USA) and found to be <100 endotoxin units/mg protein (EU/mg). The enzymatic ADP-ribosyltransferase activity was determined by the NAD:agmatine assay (38). Protein analysis was performed with SDS-PAGE, and concentrations were determined using the Bio-Rad DC protein assay (Bio-Rad, USA), according to the manufacturer's instructions.

### Nanoparticle preparation

Nanoparticles (NPLs) were produced as described by Paillard et al. (34). Briefly, maltodextrin (Roquette, France) was dissolved in 2N sodium hydroxide by magnetic stirring at room temperature. A mixture of epichlorohydrin and glycidyltrimethylammonium chloride (GTMA, a cationic ligand; both from Sigma-Aldrich, France) was added to the polysaccharide leading to the formation of a gel. After neutralization by means of acetic acid, the gel was crushed with a high pressure homogenizer (Emulsiflex C3, Avestin, Germany). The newly obtained NPLs were purified by tangential flow ultra-filtration (Centramate Minim II PALL, France) using a 300 kDa membrane (PALL, France) to remove oligosaccharides, low-molecular weight reagents and salts. Purified NPLs were freeze dried. Lyophilized NPLs were dissolved in water and a 1,2-dipalmitoyl-sn-glycero-3-phosphatidylglycerol (DPPG) lipid (Lipoid, Germany) was loaded into NPLs at a temperature above the liquid phase transition temperature of the lipid.

### CTA1-3M2e-DD and HA loading into nanoparticles

The fusion proteins or trimeric HA were loaded into premade NPL at a mass ratio 1:5 (protein:NPL), by mixing the proteins with NPLs followed by incubation for 30 min at room temperature. The recombinant extracellular domain (Met 1-Gln 528) of the hemagglutinin (HA1+HA2) was derived from Influenza A Virus H1N1 (A/Puerto Rico/8/34 virus strain) fused with a C-terminal polyhistidine tag (Sino Biological Inc., China) was resuspended in 1.98% Empigen® BB (N,N- Dimethyl-N-dodecylglycine betaine, Sigma-Aldrich, France) obtaining a protein concentration of 1 mg/ml. Then HA was incubated with either NPL or CTA1-3M2e-DD:NPL at r.t. to obtain a formulation with a mass ratio 1:5 (protein:NPL).

### Size, zeta potential, and long term stability

We determined the efficiency of protein incorporation into NPLs by native polyacrylamide gel electrophoresis (PAGE). Proteins and NPLs were dissolved in electrophoresis buffer (Tris-HCl 125Mm (pH 6.8), 10% glycerol, 0.06% bromophenol blue) and run on a 10% acrylamide-bisacrylamide gel. The gel was stained by silver nitrate to detect unbound proteins. The size and the zeta potential of the proteins and NPLs were assessed by dynamic light scattering and electrophoretic mobility with a Zetasizer nanoZS (Malvern Instruments, France). Proteins or NPLs were kept in low volume quartz batch cuvettes (ZEN2112, Malvern Instrument, France) for particle size purposes. For assessments of zeta potential samples were diluted in water to a final volume of 750 μl and loaded into a disposable folded capillary cell (DTS1070, Malvern Instrument, France). The molecular stability of CTA1-3M2e-DD (FPM2e) or the different NPLs, was assessed after 3 months, under accelerated (40°C) or standard (4°C) conditions, or after >12 months in 4°C, “sterile setting.” The molecular stability was determined by change in size or zeta potential as measured by dynamic light scattering and laser doppler velocimetry. The stability of the protein incorporated into NPLs was evaluated by native PAGE analysis, as described above. Antigen degradation was assessed by SDS-PAGE, using a denaturing buffer (Tris–HCL 125 mm (pH 6.8), 20% glycerol, 10% SDS, 2.5% β-mercaptoethanol and 0.06% bromophenol blue). The gels were stained by silver nitrate.

### *In vitro* antigen presentation assays

The D1 cell line, a long-term growth factor-dependent immature myeloid (CD11b+, CD8α-) DC line of splenic origin derived from a female C57BL/6 mouse, was generously provided by prof. P. Ricciardi-Castagnoli (University of Milan-Bicocca, Italy) (39). The D1 cells were cultured in 24-well plates (Nunc, A/S Roskilde, Denmark) in Iscove's medium (Biochrom KG, Germany), supplemented with 10% heat-inactivated fetal calf serum (Biochrom KG, Germany), 50 μM 2-mercaptoethanol (Sigma Aldrich, Sweden), 1 mM L-glutamine (Biochrom KG, Germany) and 50 μg/ml Gentamycin (Sigma Aldrich, Sweden) and stimulated for different times with 0. 2 μM of CTA1-3Eα-DD soluble protein or when incorporated into NPLs. To assess the processing efficiency of fusion protein we determined the cell surface expression of peptide plus MHC II complex by incubating D1 cells with anti-Eα(52-68):I-A^b^ complex-specific Y-Ae biotin-labeled antibody (eBiosciences, USA). Flow cytometric analysis was performed after incubation with streptavidin-APC and anti-CD11c-PE, 7AAD, MHCII-FITC at 4°C for 30 min (eBiosciences, USA). We analyzed 100,000 events using a BD-FACS LSR II instrument (BD Bioscience, USA) and the data were analyzed with FlowJo (TreeStar, USA) software.

### Antigen processing by migratory DCs and CD4^+^ T cell priming *in vivo*

Four to 6 weeks old, age, and sex-matched TCR transgenic B6.Cg-Tg(Tcrα,Tcrβ)3Ayr/J mice were immunized i.n. with 50 μg of protein using the fusion proteins alone or incorporated into NPLs. At 24 h after a single i.n administration of fusion protein or NPLs, mice were sacrificed and the mediastinal lymph nodes (mLN) were extracted and single cell suspensions were prepared. To assess the level of Eα loaded MHCII molecules on isolated migratory DCs we incubated the cells with biotin-labeled Y-Ae anti-mouse Eα(52-68):I-A^b^ Mab. In the second step we used streptavidin-APC, anti-Ly6c-BV605, anti-CD11c-BV421, anti-MHCII (I-Ab)-FITC, anti-CD11b-APC, anti-CD103-PE, 7AAD for 30 min. at 4°C (antibodies from eBiosciences, USA). We also performed adoptive transfer experiments with 2 × 10^6^ TCR transgenic CD4^+^ T cells injected i.v into recipient C57BL/6 mice after isolation using a CD4^+^ T Cell Isolation Kit (Miltenyi Biotec, Sweden). Prior to transfer the cells were stained with 5 μM CFSE and the mice were immunized i.n. with 5 μg of fusion protein alone or incorporated into NPLs. On days 2,4,6 and 8 after immunizations the mLNs were isolated and single cell suspensions were prepared followed by labeling with anti-CD3-efluor 780, anti-CD4-BV711, anti-TCR Vα2-PE, anti-TCR Vβ6-APC, and 7AAD for 30 min at 4°C (all Mabs from eBiosciences, USA). Proliferating CD4^+^TCR Vα2^+^ Vβ6^+^ cells were identified by reduced CFSE-staining. Flow cytometry analysis was performed on 500,000 events using a BD-FACS LSR II instrument (BD Bioscience, USA) and the FlowJo (TreeStar, USA) software program.

### Virus transmission and challenge experiments

Female BALB/c mice (index animals) in groups of 10 individuals were either unimmunized or immunized i.n as described above and 8 weeks later all mice were infected i.n with 3 × 10^4^ PFU H3N2 Udorn virus (A/Udorn/307/1972 (H3N2)). After 24 h these infected mice were co-housed with unimmunized uninfected DBA/2 mice (contact animals) and the level of virus transmission was determined. After 4 days the snouts and lungs of both index and contact animals were collected and viral loads were determined by the plaque assay. Briefly, tissue samples were homogenized in cold PBS using FastPrep® spheres (MP Biomedicals, Germany), and centrifuged for 10 min at 9,000 rpm at 4°C. Sample dilutions were done with OptiMEM (Thermo Fisher Scientific, USA) supplemented with 0.3% bovine serum albumin (BSA) and inoculated in 12 well plates with confluent MDCK cells and incubated for 1–2 h at room temperature. The number of plaques in the confluent cell layer was counted in the respective dilution to calculate the virus titer and then given as plaque-forming units (pfu) per ml.

Influenza virus challenge experiments were performed in groups of 10 mice at 2 weeks after the last immunization. We used a lethal i.n dose of 4 × LD50, corresponding to 2.5 × 10^3^ TCID_50_, of PR8 A/Puerto Rico/8/34 (H1N1) virus or the mouse adapted X47 virus (a reassortant between A/Victoria/3/75 (H3N2) and A/Puerto Rico/8/34 (H1N1)). Morbidity (body weight) and mortality were monitored daily for 2 weeks. Mice were sacrificed when reaching a weight loss >25–30%.

### CD4^+^ T cell immune responses

We assessed the CD4^+^ T cell response after immunizations by two different analyses. The first analysis used flow cytometry and the PE-labeled M2e-tetramer, specifically designed for the study by the NIH Tetramer Core Facility (Bethesda, USA) to identify the CD4^+^ T cells that specifically recognize and react to M2e in the context of MHC class II I-A^b^. Briefly, 2 weeks after a challenge infection lung tissue was treated with a Lung Dissociation Kit (Miltenyi Biotec Norden AB, Sweden) and single cell suspensions were prepared. Lung cells were incubated with the specific M2e-tetramer-PE and labeled with anti-CD4-Alexa700, anti-CD19-FITC, anti-F4/80-FITC, anti-CD8-APC/Cy7 Mabs and 7AAD at 4°C for 30 min (all Mabs from eBiosciences, USA). We collected 100,000 events on the BD-FACS LSR II instrument (BD Bioscience, USA) and analyzed the data using the FlowJo (TreeStar, USA) software. The second analysis used *in vitro* M2e-peptide recall responses in single cell suspensions from spleen and mLN from immunized and control mice. Briefly, 2 × 10^6^ cells/ml were cultured in plain medium or together with 1 μM of M2e peptide (Pepscan, The Netherlands) in triplicates in 96-well microtiter plates (Nunc, Denmark) in Iscove's medium (Biochrom KG, Germany), supplemented with 10% heat-inactivated fetal calf serum (Biochrom KG, Germany), 50 μM 2-mercaptoethanol (Sigma Aldrich, Sweden), 1 mM L-glutamine (Biochrom KG, Germany) and 50 μg/ml Gentamycin (Sigma Aldrich, Sweden) for 72 h at 37°C in 5% CO_2_. After 72 h we added [3H]-thymidine (PerkinElmer, USA) to the cultures for the last 6 h and [3H]-thymidine uptake was determined using a scintillation counter (Beckman, Sweden). Prior to the addition of [3H]-thymidine we collected supernatants that were stored at −80°C for further analysis of cytokine contents. We assessed IFNy and IL-17 concentrations by ELISA using 96-well plates (Dynatech Laboratories, Inc., USA) coated with 5 μg/ml of rat anti-mouse IFN-γ or IL-17 (JES5–2A5, PharMingen, USA). After washing polyclonal rabbit anti-mouse IFN-γ or anti-IL-17 antibodies (PharMingen, Denmark) at 1 μg/ml in 0.1% BSA/PBS were added to each well and the p-nitrophenyl phosphatase (Sigma Aldrich, Sweden) reaction was visualized using a Titertek Multiscan spectrophotometer (Labsystems, Sweden) at 450 nm. The concentrations of cytokines in the supernatants were expressed in pg/ml, as calculated from plotted standard curves of serial dilutions of recombinant cytokines.

### Antibody responses

Serum and BAL were collected from individual mice at indicated time points. M2e- and HA-specific IgG and IgA antibody determinations were performed by ELISA. Briefly, we used 96-well microtiter plates (MaxiSorp, Nunc, Denmark) coated with 5 μg/ml of M2e or 1 μg/ml of recombinant HA (same as described above) in 50 mM sodium bicarbonate buffer pH 9,7 and incubated overnight at 4°C. Serum or BAL were diluted 1:25 and 1:2, respectively, in 0.1% BSA/PBS and serial dilutions 1:3 in corresponding sub-wells were performed. Wells were then incubated with alkaline phosphatase-conjugated rabbit anti-mouse IgA or IgG antibodies (Southern Biotechnology, USA) at 1:1000 dilution overnight. Nitro phenyl (NPP) phosphatase substrate (1 mg/ml, Sigma Aldrich, Sweden) in ethanolamine buffer, pH 9.8, was added to each well and the reaction was read at 405 nm using a Titertek Multiscan spectrophotometer (Labsystems, Sweden). Log_10_ titers were defined as the interpolated OD-reading giving rise to an absorbance 0.4 above background, which consistently gave values on the linear part of the curve.

### Statistical analysis

Analyses of significance were done in Prism (GraphPad Software) using unpaired *t*-test. All reported *P*-values are two-sided and values of less than 0.05 were considered to indicate statistical significance. ^*^p < 0.05, ^**^p < 0.01, and ^***^p < 0.005.

## Results

### Dendritic cells effectively take up and process the combined fusion protein/nanoparticle vector

**Figure 1:**
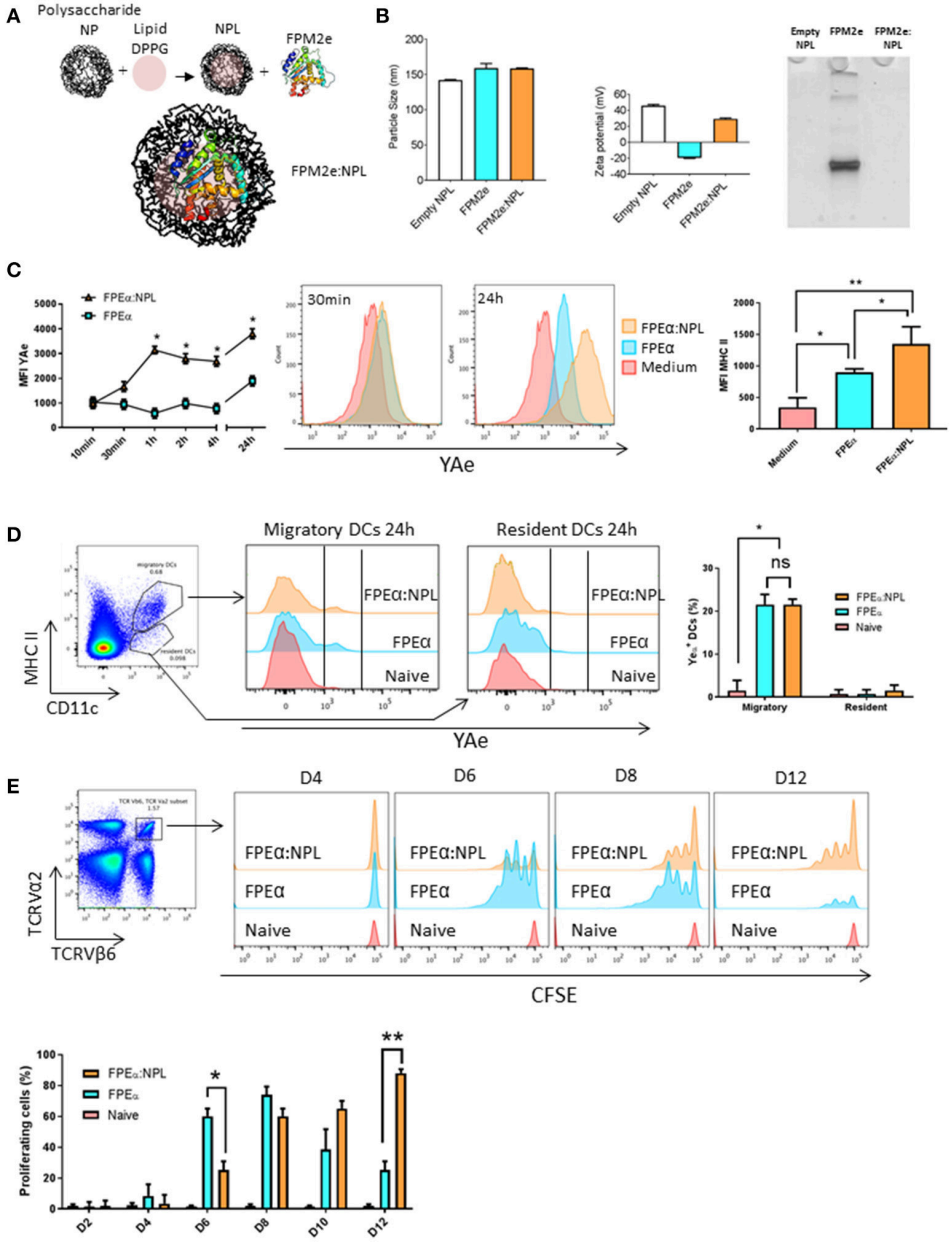
Efficient uptake and presentation of the combined NPL vaccine vector by DCs. **(A)** Schematic representation of the design of the FPM2e:NPL vaccine vector. **(B)** FPM2e:NPLs were characterized with regard to particle size (left panel), zeta potential (middle panel) and native-PAGE electrophoresis analysis (right panel). **(C)** The uptake, processing and surface presentation of Eα peptide and MHC class II complexes by D1 dendritic cells (DC) at different time points after stimulation with 0. 2 μM of FPEa or FPEa:NPL. Surface expression of peptide/MHC II complexes were analyzed by flow cytometry using the mean fluorescent intensity (MFI) of labeled Y-Ae Mab plotted as means ± SD of 3 experiments (left panel). Representative histograms of Y-Ae MFI after 30 min and 24 h stimulation are shown (middle panel). MFI values of anti-MHC II Mab labeling of the D1 cell surface after stimulation with FPEa or FPEa:NPL are given as means ± SD of 3 experiments (right panel). **(D)** Gating strategy used for migratory and resident DCs in the mediastinal lymph node (mLN) (left panel). Representative FACS histograms of Y-Ae MFI in migratory (MHC II^high^, CD11c^+^) and resident (MHC II^low^, CD11c^+^) DCs 24 h after a single i.n immunization (middle panel). The percentage of Y-Ae^+^ cells in migratory and resident DC populations was calculated in 3 independent experiments and given as means ± SD (right panel). **(E)** Gating strategy used to identify proliferation in Eα-specific CFSE-labeled TCR Tg CD4^+^ T cells following i.n immunization (left panel). Representative FACS histograms of proliferating TCRVα2^+^TCRVβ6^+^CFSE^+^ T cells in the mLN at 4, 6, 8, and 12 days after a single i.n immunization with 5 μg of FPEα or FPEα:NPL in C57Bl/6 mice adoptively transferred on day 0 with 2 × 10^6^ TCRVα2^+^TCRVβ6^+^CFSE^+^ CD4^+^ T cells (right panel). The percentage of proliferating TCRVα2^+^TCRVβ6^+^CFSE^+^ CD4^+^ T cells was calculated and given as means ± SD (lower panel). These data are from at least 3 independent experiments giving similar results. Statistical significance was calculated by unpaired *t*-test and *p*-values are given as ^*^p < 0.05 and ^**^p < 0.01.

The fusion protein CTA1-3M2e-DD (FPM2e) has previously been demonstrated to stimulate strong protective immunity against challenge with different influenza A virus subtype strains when administered intranasally (i.n) (26). However, it appeared that formulating this very effective influenza virus vaccine candidate in a suitable nanoparticle would increase its efficiency and stability as a vaccine vector even further (40). Therefore, we combined the FPM2e with porous NPLs that previously have been found effective for i.n immunizations (30, 34). Since little is known about DC uptake and presentation of antigens delivered with these nanoparticles, we initially focused on the DCs (41). A panel of formulations with different ratios between loaded protein and the NPLs was produced and their physico-chemical properties were characterized. Of this panel, we selected NPLs with a 1:5 protein:NPL mass ratio (FPM2e:NPL) as the optimal construct to be used for the continued studies. The FPM2e:NPL vector consisted of three main components: the maltodextrin scaffold (NP^+^), the lipid (DPPG) and the FPM2e, which were linked together by non-covalent interactions (Figure 1A). The FPM2e:NPL vector had an average size of 160 nm with a zeta potential of +45.63 ± 1.65 mV, i.e., highly positively charged, while the FPM2e itself was negatively charged (-19.47 ± 0.85 mV) (Figure 1B, left and middle panel). We found that most of the FPM2e had been bound to the NPLs, as shown by the absence of free FPM2e in the native PAGE analysis (Figure 1B, right panel). The combination vector was stable at different temperatures for up to 1 year with no detectable loss of FPM2e and both size and zeta-potential were kept intact (Supplementary Figures 1, 2).

To analyze antigen uptake and processing, we established an *in vitro* screening system based on NPLs carrying a fusion protein with incorporated Eα-peptide, i.e., CTA1-3Eα-DD, termed FPEα. The Eα peptide can be detected when bound to MHC class II surface molecules on DCs by using a labeled Y-Ae antibody that detects the complex (42). Therefore, the FPEα:NPL vectors were used to follow uptake and presentation of the Eα-peptide on the surface of DCs. This way, we could monitor the whole process from uptake to peptide presentation kinetically and, hence, determine what the T cell receptor would recognize on the DC surface. The initial experiments were undertaken using an immature DC cell line, D1 cells (of C57BL/6 origin), to assess the ability to present peptides to CD4^+^ T cells (39). The mean fluorescence intensity (MFI) of the bound Y-Ae antibody was assessed by FACS at different time points and from 1 h onwards we consistently observed a 2–3-fold higher MFI and also MHC class II-expression on DCs exposed to the combined vector as opposed to when the FPEα was used alone (Figure 1C, left and middle panels). Noteworthy, the CTA1-3Eα-DD given alone had a 2-fold enhancing effect on MHC class II-expression, attesting to its immunomodulating ability (Figure 1C, right panel). Thus, the combined FPEα:NPL vector was superior to soluble CTA1-3Eα-DD alone for MHC class II peptide presentation by DCs *in vitro*.

The next experiment evaluated the priming ability of DCs stimulated by FPEα:NPLs for Eα peptide-specific recognition by TCR transgenic CD4^+^ T cells (I-A^b^) *in vivo*. We used the B6.Cg-Tg(TCRα,TCRβ)3Ayr/J mice, which host TCR transgenic CD4^+^ T cells that recognize the Eα peptide bound to MHC class II. First, we determined whether the combined formulation was taken up by DCs *in vivo*. Following i.n. administration of 50 μg of the vector or soluble FPEα, we isolated the mediastinal lymph node (mLN) 24 h later and assessed the presence of DCs labeled with Y-Ae antibody (Figure 1D, left panel). We observed strong labeling with antibody in 20% of the migratory DCs (MHC II^high^, CD11c^+^) while resident DCs (MHC II^low^, CD11c^+^) did not carry the Eα-peptide and, thus, had not taken up the vaccine vector that was given i.n (Figure 1D, middle panel). Migratory DCs were found to carry the Eα peptide also when the FPEα was given i.n alone and the surface expression of the peptide/MHC II-complex was similar to that found in mice receiving the combined FPEα:NPL vector (Figure 1D, right panel). In an adoptive transfer experiment where B6.Cg-Tg(TCRα,TCRβ)3Ayr/J CD4^+^ T cells were injected into wild type C57BL/6 mice, we followed the expansion of TCR Tg CFSE-labeled CD4^+^ T cells on days 2, 4, 6, 8, 10, and 12 after the i.n immunization. We found that peptide-specific CD4^+^ T cells in the mLN, were strongly proliferating in FPEα immunized mice at the early time points, while mice given the combined FPEα:NPL vector showed similar proliferation on day 8, which was sustained until at least day 12 after immunization, when proliferation to FPEα only was minimal (Figure 1E, upper panel). Hence, peak CD4^+^ T cell proliferation to FPEα (80%) was observed on day 8 while FPEα:NPL (80%) immunized mice peaked on day 12 (Figure 1E, lower panel). Thus, the FPEα:NPL vector stimulated slower but prolonged CD4^+^ T cell activation in the draining mLN after i.n immunizations compared to that stimulated by FPEa alone.

### Enhanced immunogenicity and protective function of the combined fusion protein/nanoparticle vector

**Figure 2:**
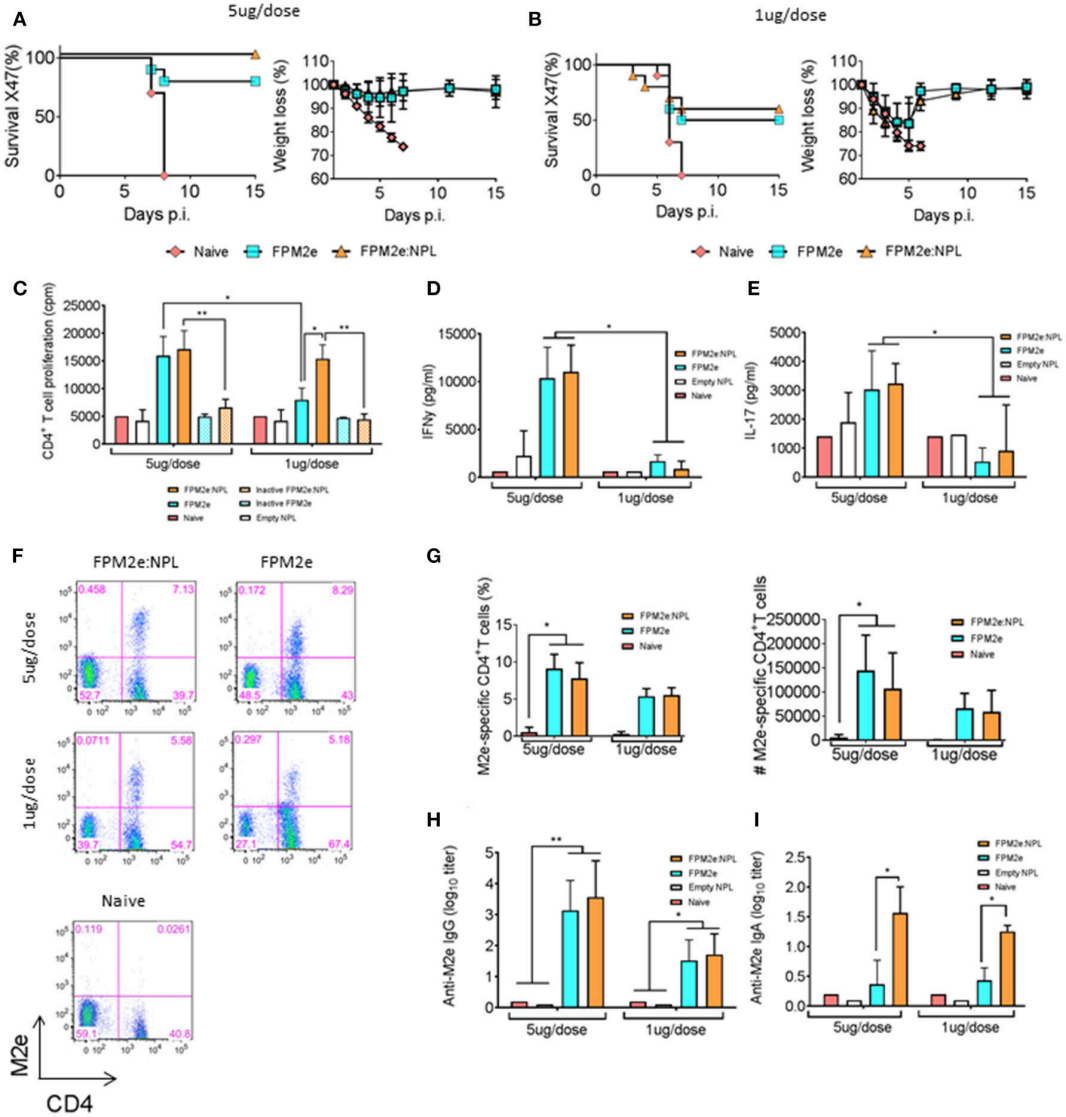
Enhanced immunogenicity of the combined NPL vaccine vector. **(A,B)** Survival and weight loss were monitored in influenza virus challenged Balb/c mice following three i.n immunizations with 5 μg **(A)** or 1 μg **(B)** of FPM2e or FPM2e:NPL. The percent of surviving mice (left panel) and body weight loss (right panel) following a challenge infection with 4 × LD50 of the mouse adapted X47 virus strain are plotted. **(C)** Recall responses to M2e-peptide in primed CD4^+^ T cells following i.n immunizations with 5 or 1 μg of enzymatically active or inactive mutant FPM2e or FPM2e:NPL or empty NPL w/o FPM2e, as indicated. Mean proliferation in isolated splenocytes to M2e peptide is given as mean c.p.m ± S.E.M. **(D,E)** The production of IFN-γ **(D)** or IL-17A **(E)** to recall stimulation with M2e-peptide of the primed CD4^+^ T cells (as in **C**) is given in pg/ml ± SD. **(F,G)** Representative FACS plots of M2e-tetramer^+^ CD4^+^ T cells in the lungs of i.n immunized and challenged mice as indicated **(F)**. The percentage (left panel) and absolute number (right panel) of antigen primed M2e^+^ tetramer CD4^+^ T cells **(G)**. **(H,I)** M2e specific IgG antibodies in serum **(H)** or IgA antibodies in BAL **(I)** were measured by ELISA in Balb/c mice immunized i.n. with FPM2e, FPM2e:NPL or PBS (naïve), as indicated, and given as mean log_10_-titers ± SD of 3 independent experiments giving similar results. Statistical significance was calculated by unpaired *t*-test and *p*-values are given as ^*^p < 0.05 and ^**^p < 0.01.

Given that the combined vector effectively primed peptide-specific CD4^+^ T cells *in vivo*, we addressed whether the FPM2e:NPL vector was also effective at stimulating protective immunity against infection. We produced FPM2e:NPL vectors with CTA1-3M2e-DD and determined their immunogenicity in BALB/c mice. Following i.n immunizations with 5 or 1 μg of FPM2e or FPM2e:NPL, we assessed the protective efficacy against a challenge with 4xLD_50_ of the X47 virus strain, a mouse adapted reassortant A/Victoria/3/75 (H3N2) virus strain (43, 44). Infected mice were monitored for weight loss and survival for 15 days post-infection. We found that mice immunized with the 5 μg/dose of FPM2e:NPL exhibited 100% protection, whereas mice immunized with FPM2e alone were less well protected (80%) (Figure 2A). Protection was clearly reduced (50%) in mice immunized with 1 μg FPM2e or FPM2e:NPL (Figure 2B). Furthermore, immunogenicity was assessed by M2e-peptide-recall responses of splenic CD4^+^ T cells isolated from immunized mice. Whereas a low dose of FPM2e:NPL (1μg/dose) was more effective than a comparable dose of FPM2e alone, both the 5 μg and 1 μg doses of FPM2e:NPL gave similar CD4^+^ T cell priming (Figure 2C). Importantly, the augmenting effect of the FPM2e:NPL formulation was dependent on the ADP-ribosylating ability of CTA1-3M2e-DD, because the combined vector with the enzymatically inactive CTA1(R9K)-3M2e-DD preparation was significantly less immunogenic (Figure 2C). The protective effect of FPM2e:NPL was associated with a strong CD4^+^ T cell priming effect for IFN-γ and IL-17 production, as assessed in culture supernatants *ex vivo* (Figures 2D,E, respectively). Finally, the presence of resident memory M2e-tetramer-specific CD4^+^ T cells in the lung was similarly high in mice immunized with the combined FPM2e:NPL vector or FPM2e alone (Figures 2F, G). In addition, strong and comparable M2e-specific antibody responses in serum were found in both FPM2e alone and the combined FPM2e:NPL vector immunized mice (Figure 2H). However, by contrast, anti-M2e IgA titers in bronchoalveolar lavage (BAL) were highest in FPM2e:NPL immunized mice, also with 1 mcg doses, clearly identifying a benefit of the NPL formulation (Figure 2I) (45, 46).

### Protection against virus transmission is effectively achieved with the combined fusion protein/nanoparticle vector

**Figure 3:**
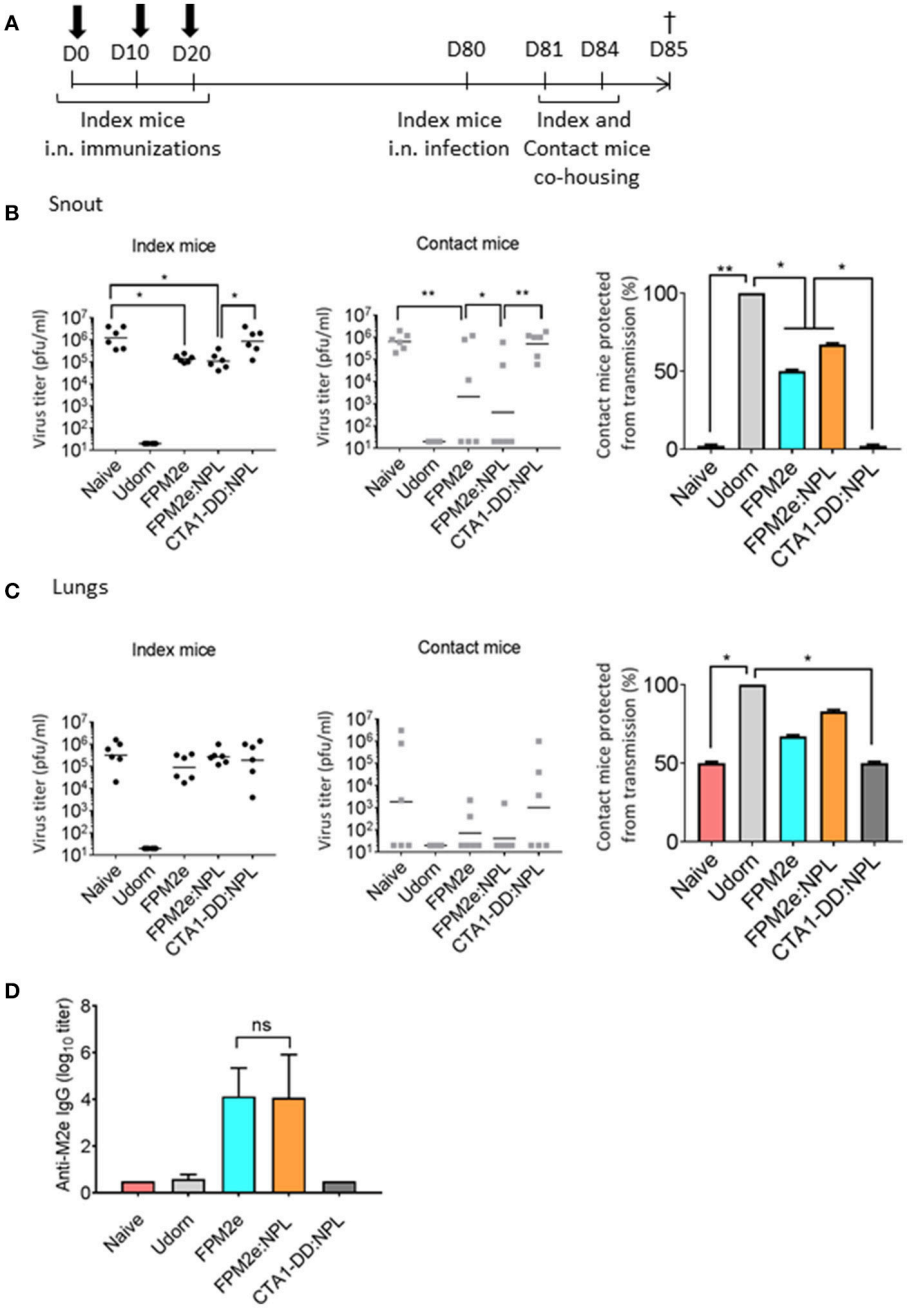
Reduction of viral transmission following intranasal immunizations with the combined NPL vaccine vector. **(A)** A schematic representation of the experimental protocol used for virus transmission experiments. BALB/c mice (index mice) were immunized three times with 10 days apart with split Udorn virus, 5 μg per dose of FPM2e alone, FPM2e:NPL, or FP:NPL w/o M2e. Index mice were infected 2–4 weeks after the final immunization with A/Udorn/307/1972 (H3N2) and co-housed with DBA/2 mice (contact mice). **(B,C)** The viral titers in snouts **(B)** or lungs **(C)** of index (left panel) and contact (middle panel) mice and the mean percentages of contact mice protected from virus transmissions (right panel) are shown. **(D)** M2e-specific IgG antibodies in serum were measured by ELISA in index mice and the r**e**sults are given as mean log_10_ titers ± SD. These are representative results from 3 experiments giving similar results and the statistical significance was calculated using unpaired *t*-test and *p*-values are ^*^p < 0.05 and ^**^p < 0.01.

An effective vaccine against influenza infection should preferentially also stop virus transmission between individuals. To this end, we tested the ability of the combined FPM2e:NPL vector to impair virus transmission between animals. We used a recently established mouse transmission model (47) with highly susceptible DBA/2 mice (48) as contact animals. Following a challenge infection with Udorn virus (H3N2) immunized and unimmunized Balb/c mice (index mice) were co-housed with the DBA/2 contact mice for 4 days (Figure 3A). Virus transmission was assessed by monitoring the influenza virus titres in the snouts and lungs of both Balb/c index and DBA/2 contact mice. We found lower virus titres in the snouts of the contact mice co-housed with index mice immunized with FPM2e:NPL (Figure 3B). However, protection against infection in the index mice was comparable between FPM2e alone and FPM2e:NPL (Figure 3B). Of note, unimmunized (PBS) mice or index mice immunized with CTA1-DD without the M2e-peptide failed to influence transmission of virus to the contact mice. The results from the analysis of the virus titers in the lungs of index or contact mice were less compelling, but also in the lung we found the least transmission from FPM2e:NPL immunized mice (Figure 3C). Anti-M2e serum antibody titers were comparable between index mice immunized with FPM2e or FPM2e:NPL (Figure 3D). Taken together the combined FPM2e:NPL vector gave the strongest protection against virus transmission, although the Balb/c index mice immunized with FPM2e:NPL or FPM2e alone exhibited comparable virus titers, suggesting that virus from FPM2e:NPL immunized mice was less infective, maybe due to local anti-M2e IgA antibodies (49).

### Co-incorporated recombinant HA improves the protective capacity of the combined fusion protein/nanoparticle vector

**Figure 4:**
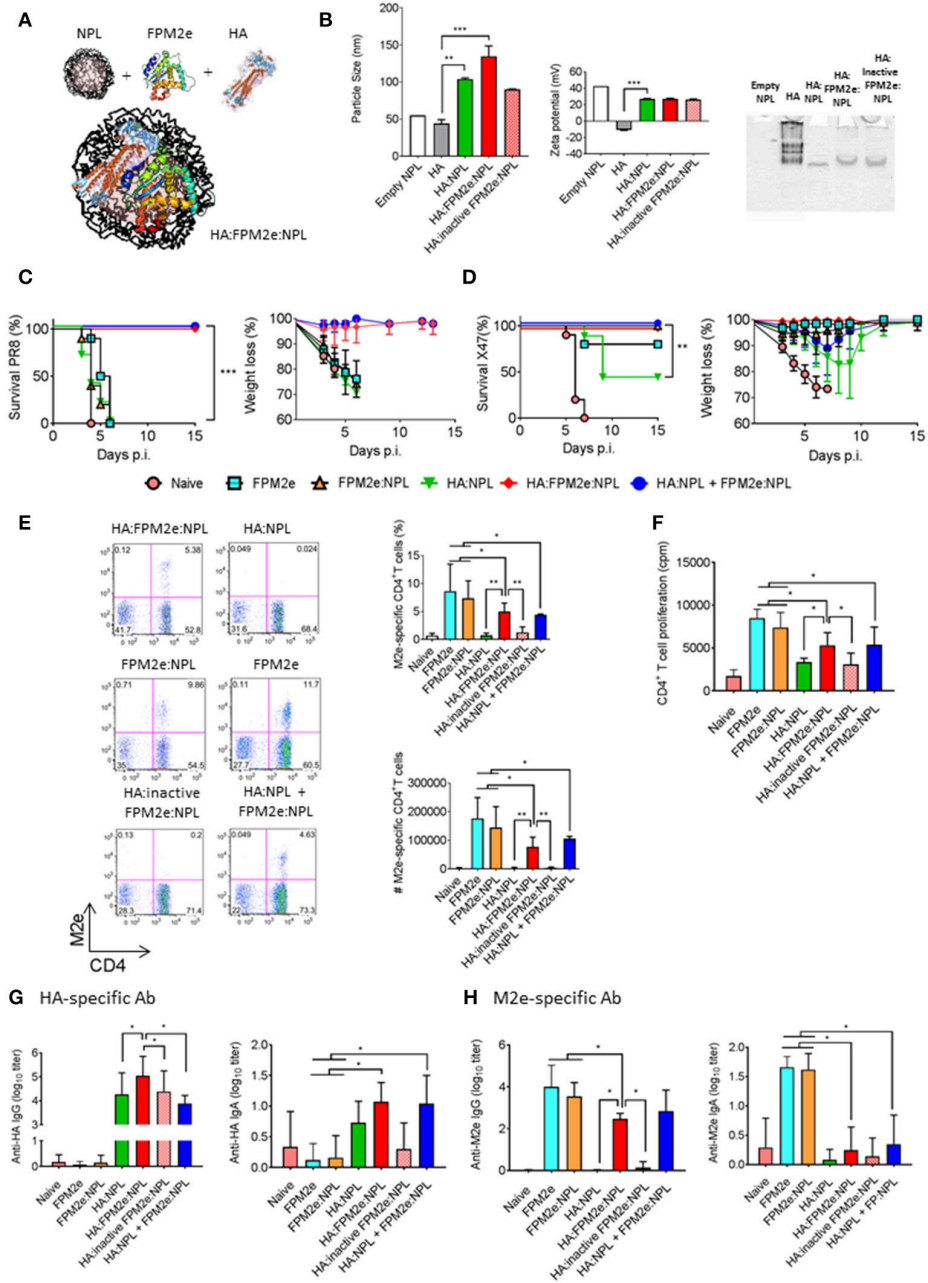
Enhanced immunogenicity and protection by co-incorporation of recombinant HA and FPM2e in the combined NPL vaccine vector. **(A)** A schematic representation of the HA:FPM2e:NPL vaccine vector. **(B)** The combined HA:FPM2e:NPL vector was characterized with regard to particle size (left panel), zeta potential (middle panel) and native-PAGE electrophoresis analysis (right panel). **(C,D)** Survival and weight loss was monitored in influenza virus challenged Balb/c mice following three i.n immunizations with 5 μg of vaccine formulations as indicated. The percent of surviving mice (left panel) and body weight loss (right panel) following a challenge infection with 4 × LD50 of the mouse adapted X47 **(C)** or PR8 **(D)** virus strains. **(E)** Representative FACS plots of M2e-tetramer^+^ CD4^+^ T cells in the lungs of i.n immunized and challenged mice are shown. The percentage and absolute numbers (right panels) of antigen primed M2e^+^ tetramer CD4^+^ T cells in the lung. **(F)** Recall responses of primed M2e-specific CD4^+^ T cells in the spleens of immunized mice are given as mean cpm± SD of 3 independent experiments. **(G,H)** HA- **(G)** or M2e-specific **(H)** IgG antibodies in serum (left panel) and IgA antibodies in BAL (right panel) were determined by ELISA in immunized mice as indicated and the mean log_10_-titers± SD are given. These are representative results from three experiments giving similar results and the statistical significance was calculated using unpaired *t*-test and *p*-values are ^*^p < 0.05, ^**^p < 0.01 and ^***^p < 0.005.

The combined FPM2e:NLP vector was found to be highly immunogenic and induced strong protection against virus transmission. However, we asked whether we could improve the protective ability of the combined vector even further by incorporating recombinant hemagglutinin (HA) from Influenza A Virus H1N1 (A/Puerto Rico/8/34) into the vector (Figure 4A). We formulated NPLs with equal amounts of CTA1-M2e-DD and HA. The HA:FPM2e:NPL vector had a size of 130 nm and a zeta potential of +27 mV (Figure 4B, left and middle panels). Noteworthy, the soluble HA protein had a particle size of around 50 nm and was negatively charged (−10 mV) (Figure 4B, left and middle panels). We found that most of the HA was incorporated into the FPM2e:NPLs (Figure 4B, right panel). Mice immunized i.n with the combined HA:FPM2e:NPL vector were fully protected against a challenge infection with the highly virulent PR8 virus (A/Puerto Rico/8/34 (H1N1), whereas none of the HA:NPL, FPM2e:NPL, or FPM2e alone immunized mice were protected (Figure 4C). Interestingly, i.n. administration of the NPL formulated CTA1-3M2e-DD (FPM2e:NPL) together with HA:NPLs still resulted in 100% protection against PR8 challenge, showing that the adjuvant CTA1 component was effective even if not physically linked to the HA:NPL (Figure 4C). By contrast, a challenge infection with the H3N2 X47 virus strain resulted in partial protection in mice immunized i.n with HA:NPL, and only to achieve 100% protection the adjuvant active FPM2e was needed (Figure 4D). As seen previously, FPM2e:NPL and FPM2e alone gave excellent protection against X47 virus infection (Figures 2A,B, 4D). Noteworthy, the frequency and absolute numbers of lung resident M2e-tetramer^+^ CD4^+^ T cells were lower in mice immunized with HA:M2e:NPL than in FPM2e:NPL or FPM2e alone immunized mice (Figure 4E). Again, we observed that the FPM2e:NPLs with an enzymatically inactive fusion protein (CTA1(R9K)-3M2e-DD) were poorly immunogenic, indicating that the performance of the NPL vector was critically dependent on the ADP-ribosylating ability of the FPM2e (Figure 4F). In fact, it was clear that the immunogenicity of the incorporated HA greatly benefitted from the adjuvant enhancing effects of the HA:FPM2e:NPL vector as anti-HA serum IgG titers were almost 10-fold higher than in HA:NLPs without the FPM2e (Figure 4G). Interestingly, though, this effect was seen only when the FPM2e was in the same particle as the HA and not when the FPM2e was co-administered in a separate NPL (Figure 4G). The M2e-specific IgG responses in serum and IgA-responses in BAL were reduced in HA-containing NPLs as compared to FPM2e:NPLs without the HA (Figure 4H). Thus, the FPM2e:NPL vector can be further improved by incorporating additional proteins into the vector and the HA:FPM2e:NPLs vaccine vector was found to exhibit superior protective capacity against a virulent PR8 influenza virus infection, where neither NPLs with HA nor CTA1-3M2e-DD gave any protection. Importantly, the immunogenicity and protective capacity of the combined HA:FPM2e:NPL vector was critically dependent on the enzymatic activity of CTA1 in the FPM2e.

## Discussion

The present proof-of-principle study demonstrates that an effective broadly protective anti-influenza mucosal vaccine vector can be developed when HA and the enzyme-active CTA1-3M2e-DD adjuvant are incorporated into NPLs. We found that the novel combined HA:FPM2e:NPL vector stimulated strong protective immune responses against homologous and heterologous infections with significantly better survival compared to mice immunized i.n with HA:NPL, FPM2e:NPL, or FPM2e alone. The vector hosted some critical features brought together in a single physical unit, namely the powerful CTA1 adjuvant, the M2e and recombinant HA for broad cross-protection and the particle formulation, facilitating mucosal delivery, stability, and uptake by DCs. These elements combined contributed to the strong protective immune response following i.n immunizations that we observed. Whereas many previous studies have reported on promising mucosal vaccine candidates against influenza, this is the first to describe the combination of an enzyme-active adjuvant system incorporated into nanoparticles (50–52). The NPL incorporation technique used did not damage the ADP-ribosylating ability of the CTA1-enzyme.

Several of the mucosal vaccine candidates against influenza that have been, or are being, tested have explored various other forms of nanoparticle formulations (53–58). Among the more successful ones are chitosan nanoparticles, that have been developed for immunizations of pigs, and which were reported to stimulate mucosal IgA as well as effector CD4^+^ T cell immunity (49, 59). Contrary to our combined vector, these nanoparticles carried multiple killed swine H1N1 antigens while we explored only the M2e-peptide and recombinant HA. However, this and several other studies support the concept of multiple influenza antigens encapsulated into nanoparticles as a promising way forward for a broadly protective influenza vaccine (60, 61). In this context, it is noteworthy that nanoparticles with killed whole-inactivated virus antigens have consistently been found to be poor inducers of T cell mediated responses and, hence, have provided only weak protection against heterologous influenza strains (62). Our study demonstrates that strong CD4^+^ T cell responses can be achieved with the present combined NPL. An explanation for the weak protection could be that injectable vaccines give poor lung resident T cell immunity, which is thought to be critical for a broadly protective influenza vaccine (4).

The porous NPL technology has been successfully used for several i.n vaccine formulations in the past, including a vaccine candidate against toxoplasma infection (31). It has repeatedly been found that the use of particulate antigens can be more effective than soluble proteins at stimulating strong immune responses and affording long-term protection (34, 35, 63–66). However, previous work with porous NPLs has not explored adding an independent adjuvant active vaccine component, such as the CTA1-3M2e-DD molecule. Here, we report that this greatly augmented the immunogenicity of the NPL vector. We observed that HA:FPM2e:NPLs achieved a 10-fold stronger anti-HA IgG serum titer when the HA was co-incorporated into NPLs with CTA1-3M2e-DD. This enhancing effect is what we regularly have observed with the CTA1-DD adjuvant in other systems (23, 24, 28). The augmenting effect required an active ADP-ribosylating enzyme, because the inactive CTA1(R9K)-3M2e-DD mutant failed to augment immunogenicity, which agrees well with results from our previous studies (67). The latter finding also identified that nanoparticles can achieve much improved immunogenicity if complemented with adjuvant active molecules, such as chitosan, flagellin or CTA1-DD (68–70). Interestingly, this augmenting effect on anti-HA IgG serum antibodies was not seen when the FPM2e and HA were provided in separate NPLs, suggesting that this effect required physical contact between HA and the FPM2e. While excellent protection was achieved also in vaccine regimens with NPLs where HA and FPM2e formulations where separated and both protocols induced comparable M2e-immunity, we can speculate that anti-HA-specific cell-mediated immunity was responsible for the improved protection against influenza virus challenge infection. We did not determine HA-specific T cell immunity in the present study, but the result is in agreement with a direct effect of the CTA1-3M2e-DD on the follicular dendritic cells (FDC) in the germinal center, which could only work if expanding HA-specific B cells were recruited to CTA1-3M2e-DD exposed FDCs (71, 72). We have recently found that this effect of the CTA1-DD adjuvant on FDCs is mediated through an up-regulation of gene transcription and, in particular, the CXCL13 gene, which encodes the main chemokine to attract activated B cells to the GC (73). However, the adjuvant effect on CD4+ T cell priming is likely to be through enhancing DC functions, which is effectively achieved with the FPM2e and would not necessarily require that HA and FPM2e are physically co-formulated in the same NPL. Additional studies are required to dissect the detailed mechanisms behind the strong adjuvanticity that we observed with the combined NPL vaccine vector.

A special focus was given to DCs for the binding and uptake of the combined NPL vector. We observed *in vitro* that Eα-peptide in the FPEα incorporated in NPLs was more efficiently taken up and/or processed and presented by DCs than when provided as soluble FPEα. The FPEα:NPL formulation gave up-regulated MHC class II expression and Eα-peptide presentation on the surface of the DCs. *In vivo*, we identified that migratory DCs were the cell subset responsible for Eα peptide priming of the specific CD4^+^ T cells in the draining mLN. With a relatively larger dose (50 μg) of FPEα:NPL than used for i.n immunizations (5 μg) we could detect Eα-presenting DC in the draining mLN. Hence, this result provides strong evidence that migratory DCs are the prime effectors of the augmented FPEα:NPL response. However, while the effect *in vitro* indicated a dramatic improvement of peptide expression in exposed DCs, the *in vivo* expression in migratory DC was comparable between FPEα alone and FPEα:NPL. The protective ability was similar between FPM2e and M2e:NPL, which was supported by comparable levels of resident M2e-specific CD4^+^ T cells in the lung and IgG-specific M2e-antibodies in serum. To reconcile these observations, it may be postulated that NPL formulations are retained in the nasal mucosa longer than the FPM2e alone and that this leads to a slower and more prolonged priming of specific CD4^+^ T cells in the mLN when FPEa:NPLs are given. Also earlier studies have observed a depot-effect and retention of CD4^+^ T cell priming in draining lymph nodes when NPL formulations were used (74). Therefore, it may be possible to improve the performance of the FPM2e:NPL vector further by altering the chemical composition of the NPL or by adding chitosan or some known component with an effect on the penetration of the mucosal barrier (72, 75–77).

*In vivo*, we found that a higher M2e-specific CD4^+^ T cell priming effect was achieved with the lower FPM2e:NPL dose as compared to an equivalent FPM2e dose. This was evident from recall responses to M2e-peptide in isolated splenocytes from immunized mice. Strong support for the requirement of an active ADP-ribosylating activity of the CTA1 enzyme was also found in these experiments. The enhanced response showed augmented levels of IFN-γ and IL-17 production from the M2e-specific CD4^+^ T cells, which are the cardinal features of strong heterosubtypic protection in the mouse model of influenza infection (78–82). We could identify the presence of lung resident M2e tetramer-specific CD4^+^ T cells in these well protected mice, which confirms the pattern that we previously identified with the FPM2e and places emphasis on the very important role of these tissue resident M2e-specific CD4 T cells for heterosubtypic protection (26). Also, M2e-specific IgA antibody titers in BAL were higher in mice immunized with the FPM2e:NPL. However, because both FPM2e alone and FPM2e:NPL induced protection against virus transmission in immunized mice, albeit slightly better in FPM2e:NPL mice, the protective role of IgA anti-M2e antibodies is not clear. Other studies, such as that from Hervé et al, have suggested that an enhanced anti-M2e IgA antibody response after i.n immunizations could be protective (49). Noteworthy, though, is the fact that IgA antibodies are likely not mediating ADCC reactions and, hence, the role of local respiratory tract anti-M2e IgA for protection is at present poorly defined. Nonetheless, mucosal IgA anti-HA following i.n immunizations with in HA:FPM2e:NPL may well play a protective role, as suggested in several other studies (52, 83, 84). In addition, our recent study with M2e-specific lung resident memory CD4^+^ T cells has clearly pointed to a critical protective function of these cells, which are only generated after i.n immunizations (26). Hence, the co-existence of local IgA and influenza-specific resident memory CD4^+^ T cells makes it difficult to identify the relative contribution each of these elements for protection, but ongoing studies in our laboratory is attempting to better dissect this question (85, 86).

In the present study we have convincingly shown that co-incorporation of adjuvant active molecules and influenza specific target antigens into porous NPLs is more broadly effective against influenza virus infections than either component used alone. Hence, we would like to continue developing the NPL vector with additional components known to exert broad protection against influenza. In particular, we will test the addition of the nucleoprotein (NP), which can elicit strong cytotoxic CD8^+^ T cells (4). In addition, instead of whole recombinant HA, we propose to include a stabilized HA stem region, as recently reported using ferritin nanoparticles, which stimulated protection against a heterosubtypic challenge infection in both mice and ferrets (5, 66). In addition, we noticed that the presence of recombinant HA in the NPL formulation significantly reduced the anti-M2e antibody and CD4^+^ T cell responses, suggesting that we need to increase the FPM2e component in future combined NPL vectors. This way we may also achieve improved adjuvanticity for HA-immune responses. Future studies will reveal if the favorable effects observed with the combined HA:FPM2e:NPL i.n vaccine vector for broad protection against influenza can be translated into a human vaccine.

## Ethics statement

This study was carried out in accordance with the recommendations of PREPARE (Planning Research and Experimental Procedures on Animals: Recommendations for Excellence), Jordbruksverket. The protocol was approved by Jordbruksverket, the Swedish Board of Agriculture.

## Author contributions

VB, XS, PS, DB, and NL designed the study, analyzed data and wrote the manuscript. BB, ML, and RC prepared the formulations. VB, LY, AO, and KS performed the experiments and analysis of the data.

### Conflict of interest statement

The authors declare that the research was conducted in the absence of any commercial or financial relationships that could be construed as a potential conflict of interest.

## Supplementary material

Supplementary Figure 1 FPM2e:NPL vaccine vectors are stable up to 12 months.**(A)** Characterization of the stability of FPM2e:NPL by native PAGE analysis after 3 months of storage at 4°C (left panel) or 40°C (middle panel) or after 12 months at 4°C (right panel). **(B)** Characterization of the stability of FPM2e:NPL by SDS-PAGE analysis after 3 months of storage at 4°C (left panel) or 40°C (middle panel) or after 12 months at 4°C (right panel). These are representative experiments out of 3 with similar results.

Supplementary Figure 2 Size and charge of FPM2e:NPL is stable after 3 months storage. (**A)** Characterization of the size stability of FPM2e:NPLs after 3 months of storage at 4°C (left panel) or 40°C (right panel). **(B)** Characterization of the charge stability of FPM2e:NPL after 3 months of storage at 4°C (left panel) or 40°C (right panel). These are representative experiments out of 3 with similar results.
