# Supplementary figures and images for: Porous Nanoparticles With Self-Adjuvanting M2e-Fusion Protein and Recombinant Hemagglutinin Provide Strong and Broadly Protective Immunity Against Influenza Virus Infections
Source: Front Immunol. 2018 Sep 12;9:2060. doi: 10.3389/fimmu.2018.02060 (PMC6146233; doi:10.3389/fimmu.2018.02060)

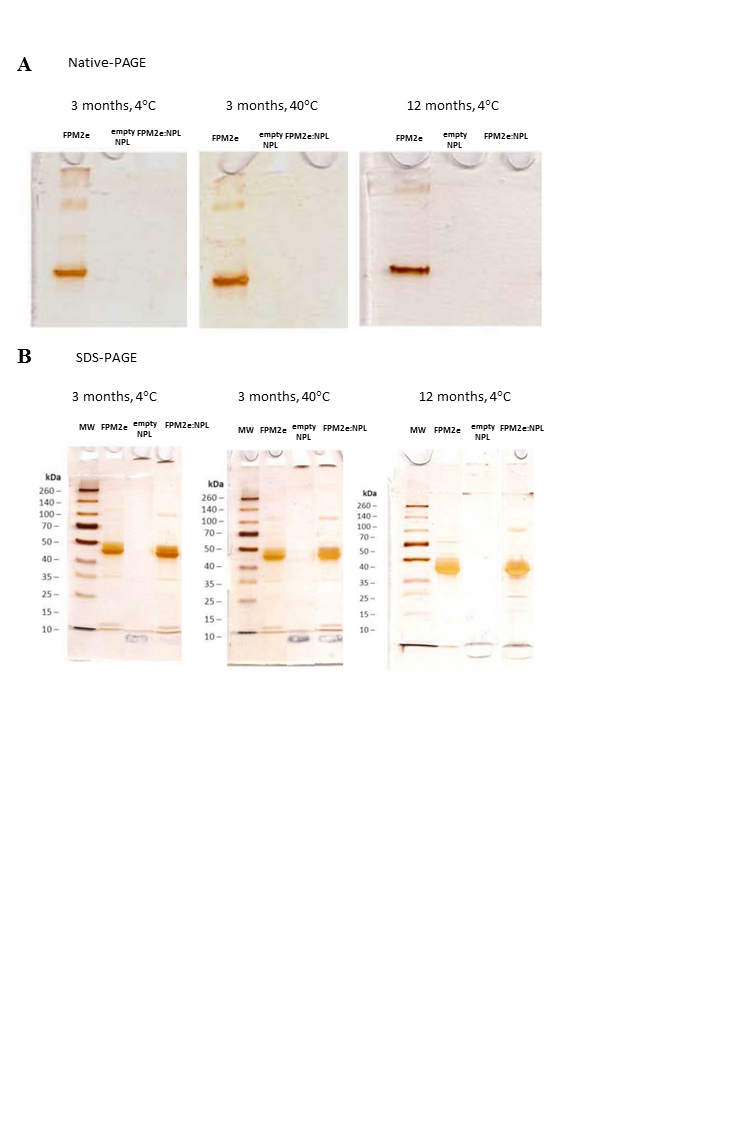

Supplement: Supplementary Figure 1 — FPM2e:NPL vaccine vectors are stable up to 12 months.(A) Characterization of the stability of FPM2e:NPL by native PAGE analysis after 3 months of storage at 4°C (left panel) or 40°C (middle panel) or after 12 months at 4°C (right panel). (B) Characterization of the stability of FPM2e:NPL by SDS-PAGE analysis after 3 months of storage at 4°C (left panel) or 40°C (middle panel) or after 12 months at 4°C (right panel). These are representative experiments out of 3 with similar results. [file Image_1.TIF]

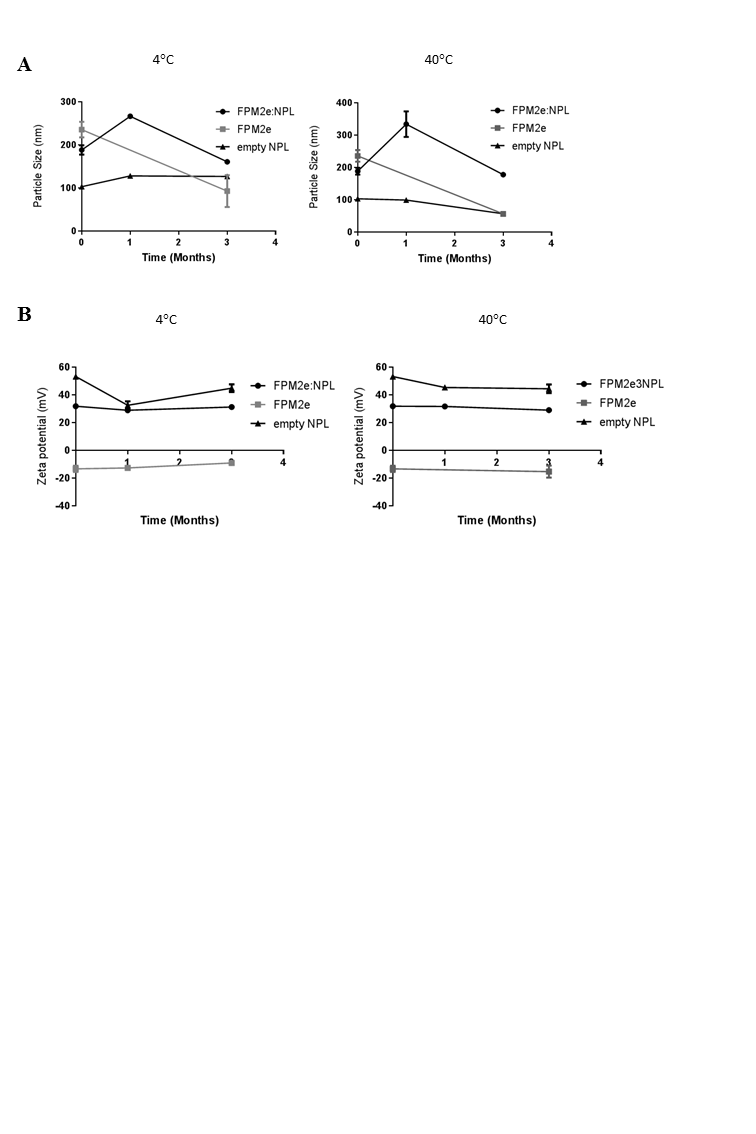

Supplement: Supplementary Figure 2 — Size and charge of FPM2e:NPL is stable after 3 months storage. (A) Characterization of the size stability of FPM2e:NPLs after 3 months of storage at 4°C (left panel) or 40°C (right panel). (B) Characterization of the charge stability of FPM2e:NPL after 3 months of storage at 4°C (left panel) or 40°C (right panel). These are representative experiments out of 3 with similar results. [file Image_2.TIF]
